# Reduction of Polyunsaturated Fatty Acids with Tumor Progression in a Lean Non-Alcoholic Steatohepatitis-Associated Hepatocellular Carcinoma Mouse Model

**DOI:** 10.7150/jca.48495

**Published:** 2020-07-20

**Authors:** Elizabeth M Vlock, Shilpa Karanjit, Geoffrey Talmon, Paraskevi A Farazi

**Affiliations:** 1 Department of Epidemiology, College of Public Health, University of Nebraska Medical Center, Omaha, NE, USA.; 2 Nebraska Center for the Prevention of Obesity Diseases through Dietary Molecules, College of Education and Human Sciences, University of Nebraska Lincoln, Lincoln, NE, USA.; 3 Department of Pathology and Microbiology, College of Medicine, University of Nebraska Medical Center, Omaha, NE, USA.

**Keywords:** liver cancer, non-alcoholic fatty liver disease, unhealthy diet, sugar, fat

## Abstract

**Background and Aim:** Non-alcoholic fatty liver disease (NAFLD) is the most common liver disease in Western countries. While obesity and diabetes are the hallmarks of NAFLD, it also develops in lean individuals in the absence of metabolic syndrome, with a prevalence of 7 percent in the U.S. and 25-30 percent in some Asian countries. NAFLD represents the spectrum of liver disease*,* starting with excess liver fat accumulation (NAFL) that can progress to nonalcoholic steatohepatitis (NASH), cirrhosis and ultimately hepatocellular carcinoma (HCC). To date, the pathogenesis of lean NASH-HCC is poorly understood and a mouse model is lacking. We aimed to develop a mouse model of lean NASH-HCC using a choline deficient and high trans-fat/sucrose/cholesterol diet to enable better understanding of its molecular pathogenesis.

**Methods:** C57BL/6N mice were fed this diet starting at 4 weeks of age for 52 weeks and were compared to mice fed an isocaloric low fat control diet for the same duration. C57BL/6N mice were chosen instead of the C57BL/6J mice due to the high susceptibility of C57BL/6J mice to diet-induced obesity. The plasma and tumor fatty acid profile of these mice was also investigated.

**Results:** Nearly 61% of the mice developed lean NASH-HCC. These mice showed reduction of plasma polyunsaturated fatty acids (PUFAs) (linolenic acids (α and γ, ω-3 and ω-6, respectively), eicosapentanoic acid (ω-3), docosahexanoic acid (ω-3), and linoleic acid (ω-6)) and increasing levels over time in mice with pre-malignant lesions.

**Conclusions:** We developed a novel high penetrance diet-induced lean NASH-HCC mouse model. Plasma PUFA levels were reduced with tumor progression in parallel with reduced expression of genes controlling desaturase expression suggesting their potential use as biomarkers for lean NASH-HCC progression as well as chemopreventive molecules.

## Introduction

NAFLD has a 24 percent prevalence globally and is predicted to be the leading cause for liver transplantation in the United States by 2030 1,2. It represents the spectrum of liver disease*,* starting with excess liver fat accumulation (NAFL) that can progress to nonalcoholic steatohepatitis (NASH), cirrhosis and ultimately hepatocellular carcinoma (HCC) 3. NAFLD accounts for an increasing number of HCCs 4,5,6.

While obesity, diabetes, and genetic predisposition are the hallmarks of NAFLD, it also develops in lean individuals in the absence of metabolic syndrome, with a prevalence of 7 percent in the U.S. and 25-30 percent in some Asian countries 2. Lean NAFLD is caused by genetic mutations, resulting in triglyceride accumulation in the liver 7, or, more typically, by diet-related visceral obesity (diet high in fructose, fat, and cholesterol). Lean NAFLD patients differ from obese NAFLD patients in that they do not exhibit all the co-morbidities of metabolic diseases, such as insulin resistance, higher serum cholesterol and triglyceride levels, or higher liver enzymes. Lack of typical risk factors makes identification of NAFLD in lean patients difficult, thus increasing the likelihood of delayed diagnosis and poor prognosis 8,9.

Lipogenesis and changes in lipid metabolism are characteristic in NAFLD progression. Alterations in fatty acids in the serum of NASH patients have been reported 10,11. Currently studies have not addressed the progression to HCC. However, it has been shown that various phospholipids and ceramides are reduced in human HCC tissues compared to adjacent tissues 12. Furthermore, specific patterns of serum and tissue fatty acid levels were shown to correlate with early-stage HCC in a genetic murine model of NASH 13 and the levels of free fatty acids were elevated in the livers of mice with diet-induced NAFLD 14.

Various mouse models of diet-induced HCC have been generated. However, these mouse models develop diet-induced HCC in the context of obesity and/or they develop HCCs with low penetrance or with high penetrance after a very long duration of feeding (80 weeks) 15,16,17,18. To date, there is no model of lean NASH-HCC with high penetrance that would allow further investigation into the molecular mechanisms of this disease. To this end, we sought to develop a lean NASH-HCC mouse model by utilizing a choline deficient, high trans-fat/sucrose/cholesterol diet to induce NASH-HCC in mice of the C57BL/6N strain. We further investigated plasma and tissue levels of fatty acids to characterize changes in lipid metabolism during disease progression.

## Materials and Methods

### Animals and experimental diets

This study was conducted with the approval of the Institutional Animal Care and Use Committee at the University of Nebraska Medical Center (UNMC) (Protocol #: 16-121-11). Male C57BL/6N mice (*n* = 50) were obtained from Charles River Laboratories at 3 weeks of age. Upon arrival they were allowed to acclimate for a week and at 4 weeks their diet was switched from the standard chow diet to refined diets. They were housed in a temperature-, humidity-, and ventilation-controlled vivarium and kept on a 12-h light/dark cycle under specific pathogen-free conditions. 35 mice were fed a choline deficient, high trans-fat, sucrose, and cholesterol diet (CD-HFSC; D16120210, Research Diets, New Brunswick, New Jersey). 15 mice were fed a low-fat control diet (D16120211, Research Diets, New Brunswick, New Jersey) (**Supplemental Table S1**). Food consumption was monitored twice a week by recording weight of feed before and after changing feed.

### Mouse health surveillance

Mice were weighed weekly and regular husbandry checks were performed for signs of anemia, jaundice, weight loss, and dermatitis. Mice were euthanized if they appeared sick per institutional guidelines. Ultrasound evaluation was performed at 46 weeks of age using the VisualSonics Vevo 3100 ultrasound system (Fujifilm, VisualSonics, Toronto, ON) with the MX550D transducer (40 MHz center frequency, 40 µM axial resolution). In brief, mice were anesthetized using 1-3% isoflurane, placed on a heated monitoring table, and had abdominal hair removed. B-mode images and 3D volumes were acquired in the abdominal area where liver nodules were investigated. Vevo Lab software was used to reconstruct nodule shapes and to measure nodule volumes. Nodules that measured 1mm × 1mm were considered false positives.

### Glucose tolerance test

Intraperitoneal glucose tolerance tests (IPGTT) were performed at 8, 20, 32, and 44 weeks of age. Five mice from each diet group were randomly selected and tested at 8 and 20 weeks and all mice were tested at 32 and 44 weeks. Mice were fasted for 6 hours in the morning, weighed and then glucose (2 g/kg) was administered through intraperitoneal injection. Blood samples were taken from the tail vein and glucose levels were measured with Nova Max Plus glucometer and test strips (Waltham, Massachusetts) prior to glucose injection (0 minutes), and at 15, 30, 60, and 120 minutes post glucose challenge. Glucose levels were graphed as a function of time and the area under the curve was calculated by the trapezoid method 19.

### Measurement of plasma lipids and liver enzymes

Whole blood was collected from CD-HFSC mice (*n* = 31) and control diet mice (*n* = 15) at 26 weeks of age to assess plasma lipids (cholesterol, triglycerides) and liver enzymes (alanine aminotransferase, ALT; aspartate aminotransferase, AST). Approximately 300 µL of blood was collected in lithium heparin microtubes from the mandibular vein after a 6 hour morning fast. Blood samples were centrifuged at 13,000×g for 5 minutes at 4 °C. Plasma was aliquoted and stored at -80 °C until processing. Approximately 100 µL of plasma was analyzed using the Vitros-250 system (Ortho-Clinical Diagnostics, Linden, New Jersey) to measure the levels of ALT, AST, triglycerides, and total cholesterol at the Nebraska Center for Prevention of Obesity Diseases (NPOD) Biomedical and Obesity Research Core (BORC) in Lincoln, Nebraska.

### Histological Evaluation

Livers were excised post mortem, weighed, and observed grossly for the appearance of nodules. Parts of tissues were snap frozen in liquid nitrogen and stored at -80 °C and the remaining tissues were fixed in 10% formalin for 2 hours and paraffin embedded (UNMC Tissue Sciences Facilities). Tissue sections were stained with Hematoxylin and Eosin (H&E), Masson-Trichrome, and reticulin and were assessed blindly by an experienced pathologist for steatosis, ballooning, and inflammation. NAFLD and NASH were assessed using a previously described scoring system 20. The stained liver sections were also evaluated for the presence of regenerative nodules, dysplastic nodules, and hepatocellular carcinomas. Regenerative nodules were apparent by H&E stain, did not compress the surrounding hepatocytes, and had an intact reticulin network surrounding the hepatocytes with mild cytologic atypia (cell change). Dysplastic nodules were obvious on low power by H&E stain, were expansile in nature, and compressed the surrounding hepatocytes with limited destruction of reticulin network. Nodules were classified as HCC if they were expansile and compressed the surrounding hepatocytes with destruction of the reticulin framework and pseudo acinar changes and/or cytologic atypia.

### Metabolomics

Plasma collected at 32 and 55 weeks of age and tissue collected at 55 weeks of age were analyzed for fatty acid levels. Plasma samples were randomly selected from mice fed the CD -HFSC diet that had HCC (*n* = 5), mice with regenerative or dysplastic nodules without HCC (pre-malignant nodules; *n* = 5), and HCC-free mice fed the control diet with NAFLD (*n* = 5). Five tissue samples from HCCs obtained from the same mice were also analyzed.

Plasma (*n* = 30) and tissue (*n* = 5) samples were prepared for analysis through dilution and homogenization of tissue samples in a phosphate buffer using a bead homogenizer (Precellys, Bertin Technologies). Samples were adjusted to a final volume of 0.5-1 mL with LC-MS grade water, acidified with dilute hydrochloric acid and extracted with isooctane-ethyl acetate. Extracts were then dried and reconstituted in methanol-water-ammonium acetate. The extracts were sent to the Lipidomics Core Facility at Wayne State University for fatty acid metabolomics analysis. The extracts were subjected to HPLC on Targa C8 column and directly introduced to the QTRAP5500 mass analyzer (ABSCIEX). The data were collected using Analyst 1.6.2 software and the chromatograms were quantitated by MultiQuant software (ABSCIEX). In total 30 fatty acids (C12-C26) were analyzed (**Supplemental Table S2**).

### mRNA sequencing analysis

Total RNA was extracted from liver tissue of mice fed the control diet (2 mice with NAFL) and those fed the CD-HFSC diet (3 mice with HCC). RNA was isolated from approximately 10 mg of snap-frozen liver tissue using Qiagen RNeasy lipid tissue kit (Qiagen, Cat No 74804, Germantown, MD). Purity and concentration were assessed by measurement of the A260/280 ratios using a Nanodrop (Thermo Scientific, Nanodrop Products, Wilmington, DE) spectrophotometer and only those samples with values of 1.8 to 2.0 underwent further processing. Samples were sent to BGI (Cambridge, MA) for degradation testing via analysis of 200ng of RNA with a Bioanlayzer instrument (Aligent Technologies, Santa Clara, CA) and only intact RNA samples were utilized to make sequencing libraries. Libraries were sequenced using BGI's DNBseq platform which utilizes the BGISEQ-500 sequencing system and provides coverage of approximately 20 million reads per sample in 50bp single end reads. Following sequencing bioinformatics were conducted by BGI. Briefly, sequences were trimmed for quality control and aligned to the mouse genome. Differential expression was determined using RNA-Seq by Expectation-Maximization (RSEM; https://deweylab.github.io/RSEM/) and a false discovery rate of ≤0.01 were used as significant selection criteria.

### Western blotting

Tissue lysates were prepared using a standard protein extraction protocol from 3 mice with NAFLD (control diet fed group), 2 mice with NASH, 3 mice with premalignant nodules and 3 mice with HCC (CD-HFSC fed group). Equal amounts of protein (20 µg) were run on SDS-PAGE on a 4-12% Bis-Tris NuPage Gel (ThermoFisher Scientific, Waltham, MA) then transferred to nitrocellulose membranes and incubated with primary antibodies: 1) PPARα (ab24509; Abcam) at 1:1000 dilution and 2) β-actin HRP (#4970, Cell Signaling Technology) at 1:1000 dilution. Membranes were incubated with appropriate horseradish peroxidase-conjugated secondary antibodies (anti-rabbit, IgG #7074S; Cell Signaling Technology). Bound antibody was visualized on X-ray film. Membranes were stripped using Restore Western Blot Stripping Buffer (ThermoFisher Scientific, Waltham, MA) for incubation with the second antibody.

### Statistical Analyses

Data analyses were performed using SAS 9.2 software (SAS Institute Inc, Cary, North Carolina). Continuous data were expressed as mean +/- standard deviation or as absolute number or percentage for categorical variables. Group comparisons were performed using a Student's two-tailed t-test and comparisons of AUC were analyzed with repeated-measures ANOVA. For differences in fatty acid levels, a one-way ANOVA was performed and significance determined using Tukey's post hoc test. A p value < 0.05 was considered statistically significant for all comparisons.

## Results

### Assessment of weight gain and glucose tolerance

**Figure 1:**
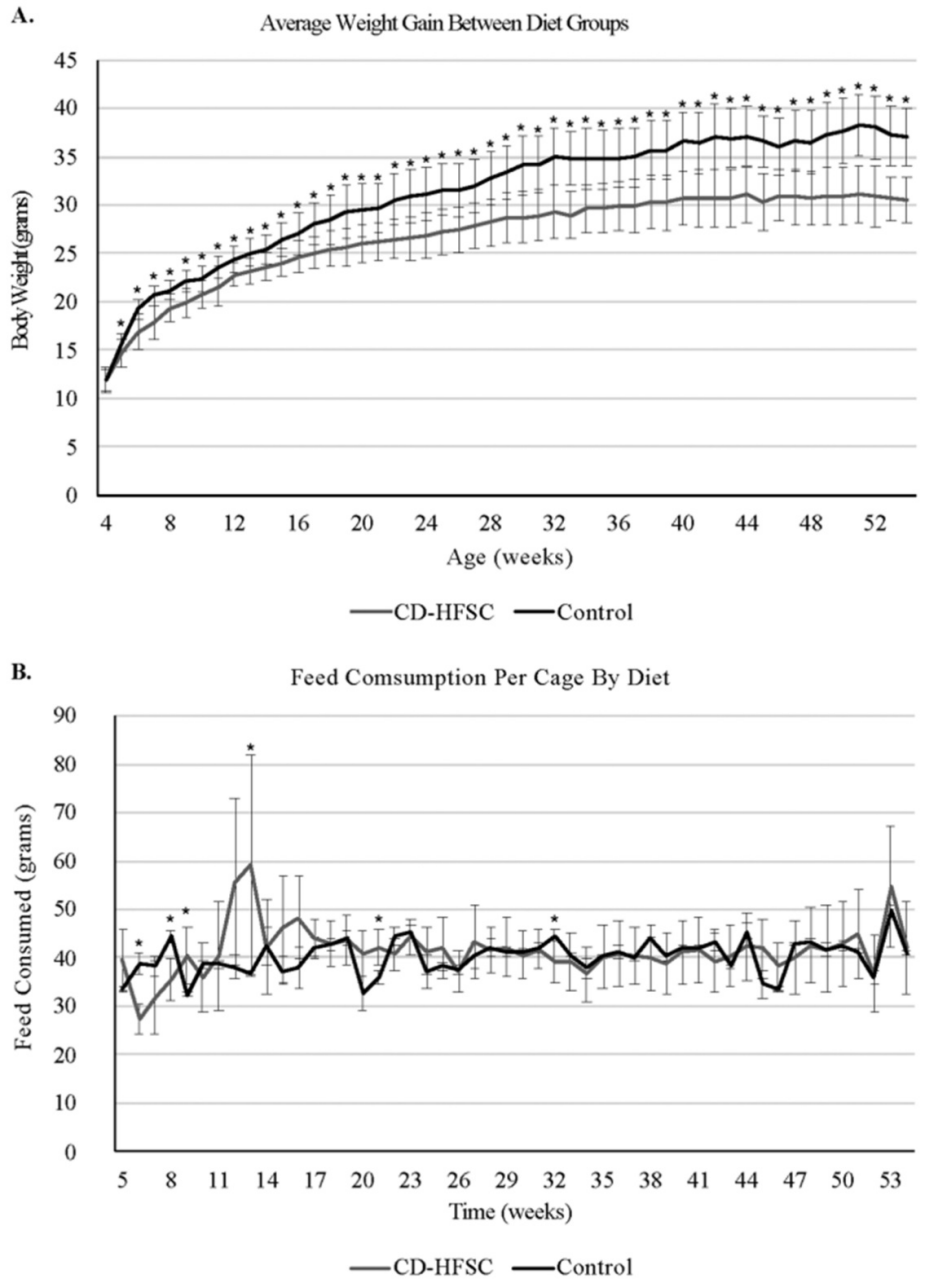
Weight gain and food consumption. **A.** Weight of mice fed the control diet (black) and the CD-HFSC diet (grey). Mice fed the CD-HFSC diet gained weight but had lower weight compared to mice fed the control diet (P < 0.001 after week 4). **B.** Feed consumption was assessed per cage. Overall the two groups of mice seem to be consuming the same amount of food. * = p < 0.05.

**Figure 2:**
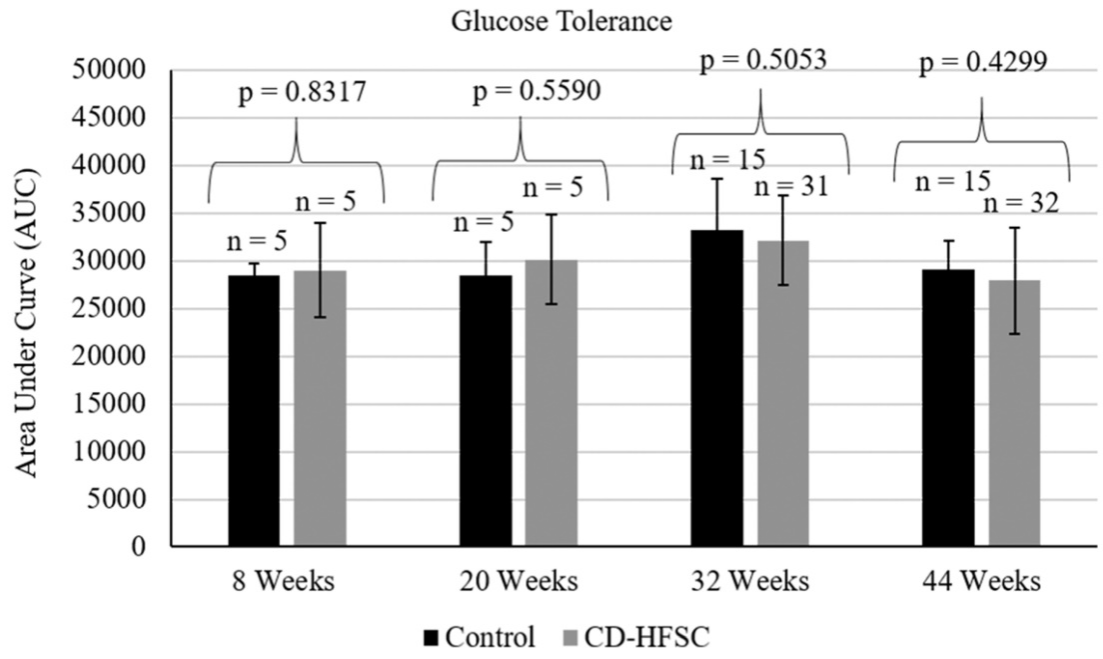
Glucose tolerance tests in mice fed the control diet (black) and the CD-HFSC diet (grey). Mice fed the CD-HFSC diet show no difference in glucose tolerance compared to mice fed the control diet.

Mice on the CD-HFSC diet did not become obese and gained significantly less weight than mice fed the low-fat control diet (p = 1.74 × 10^-6^) (**Figure 1A**) even when on average consuming the same amount of food (p = 0.293) (**Figure 1B**). CD-HFSC and control mice gained an average of 155% (+/- 32.89%) and 215% (+/- 46.8%) of their starting weight respectively (data not shown). Glucose tolerance was measured every 12 weeks starting at 8 weeks of age until 44 weeks. There were no pronounced differences between mice fed the CD-HFSC diet and mice fed the low-fat control diet (**Figure 2**).

### Assessment of liver damage and plasma lipid profile

**Figure 3:**
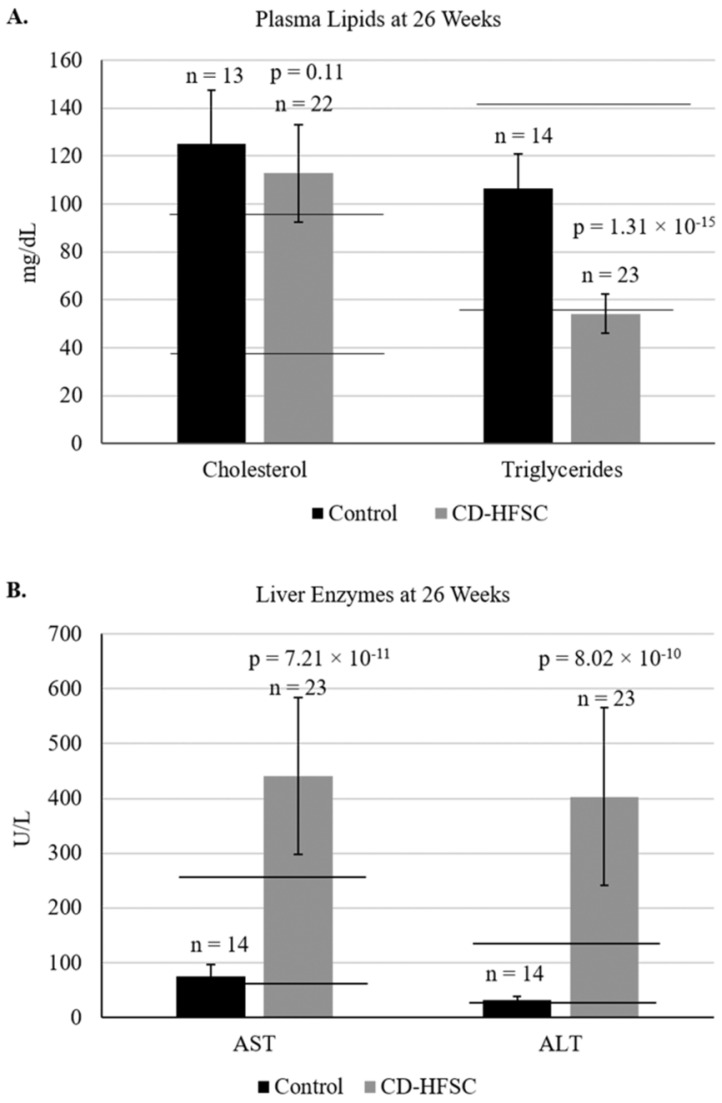
** A.** Plasma lipid and **B.** plasma enzyme profile in mice fed the control diet (black) and the CD-HFSC diet (grey). Mice fed the CD-HFSC diet show lower plasma triglyceride levels and higher levels of plasma aspartate aminotransferase (AST) and alanine aminotransferase (ALT) compared to mice fed the control diet, likely reflecting lipid retention in the liver and liver damage, respectively. Horizontal black lines indicate lower and upper limits of normal plasma lipid and enzyme levels.

Blood was collected from mice at 26 weeks of age to analyze liver enzymes (AST, ALT), and plasma lipids (cholesterol and triglycerides). Plasma levels of cholesterol were similar in mice of both diet groups; however, triglyceride levels were lower in mice fed the CD-HFSC diet compared to mice fed the control diet. Cholesterol levels in both diet groups were higher than the normal range of plasma cholesterol (**Figure 3A**). Both ALT and AST liver enzyme levels were significantly higher in mice fed the CD-HFSC diet compared to mice fed the control diet (**Figure 3B**).

### Assessment of NASH and HCC development

Mice fed the CD-HFSC diet had enlarged livers, significantly higher liver to body weight ratio and enlarged spleens compared to mice fed the control diet. Mice fed the CD-HFSC diet developed an average of 11 nodules with an average size of 44.20 mm^2^. None of the mice fed the control diet developed any nodules (**Supplemental Table S3**). Ultrasound evaluation of 10 mice (CD-HFSC *n* = 5; Control *n* = 5) at 46 weeks revealed nodules with an average size of 8.28 mm^2^ in 100% of CD-HFSC fed mice evaluated but no nodules in mice fed the control diet (data not shown).

**Figure 4:**
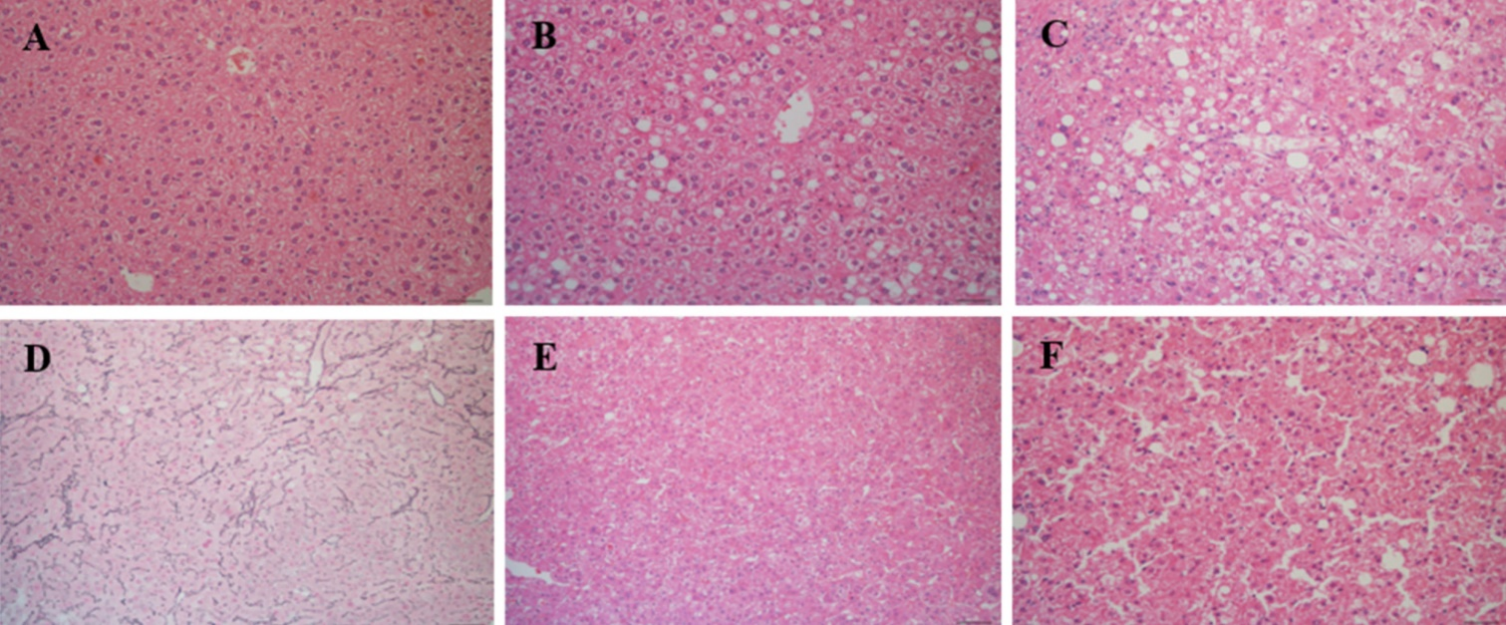
Mice fed the CD-HFSC diet developed NASH-HCC with high penetrance by 55-56 weeks of age. **A.** H&E stain of normal hepatic tissue; **B.** NAFL; H&E stain displaying some fat with little inflammation or hepatocyte damage; **C.** NASH; H&E stain with inflammation and hepatocyte damage with fat in liver; **D.** regenerative nodule compressing surrounding parenchyma with an intact reticulin network; **E.** H&E stain of dysplastic nodule compressing hepatocytes; **F.** H&E stain of HCC compressing surrounding hepatocytes with focal destruction of reticulin framework.

Liver tissue was analyzed in all mice at time of euthanasia. In total, 28 CD-HFSC mice (100%) developed NASH while 7 (46.67%) normal diet mice had normal phenotypes and 8 (53.33%) had NAFL (**Figure 4A-C**). No mouse fed the control diet developed NASH. Of the 28 CD-HFSC fed mice 13 (46.4%) had a ballooning score of 1, and 15 (53.6%) had a ballooning score of 2 (0-2 scale). In addition, the liver tissue was assessed for fibrosis using Trichrome stains. In total, 14 (50%) of the 28 CD-HFSC mice had a fibrosis score of 1, while 12 (42.8%) had a score of 2 and 2 (7.1%) had a score of 3 (**Table 1**). None of the control mice had fibrosis. Regenerative, dysplastic, and HCC nodules were also observed (**Figure 4 D-F**). Mice fed the CD-HFSC diet developed HCC with high penetrance (60.7%). In addition, 92.9% and 89.3% mice fed the CD-HFSC diet developed regenerative nodules and dysplastic nodules, respectively (**Table 1**). None of the control mice developed any type of nodules.

### Metabolic profile of fatty acids in plasma and HCC tissues

**Figure 5:**
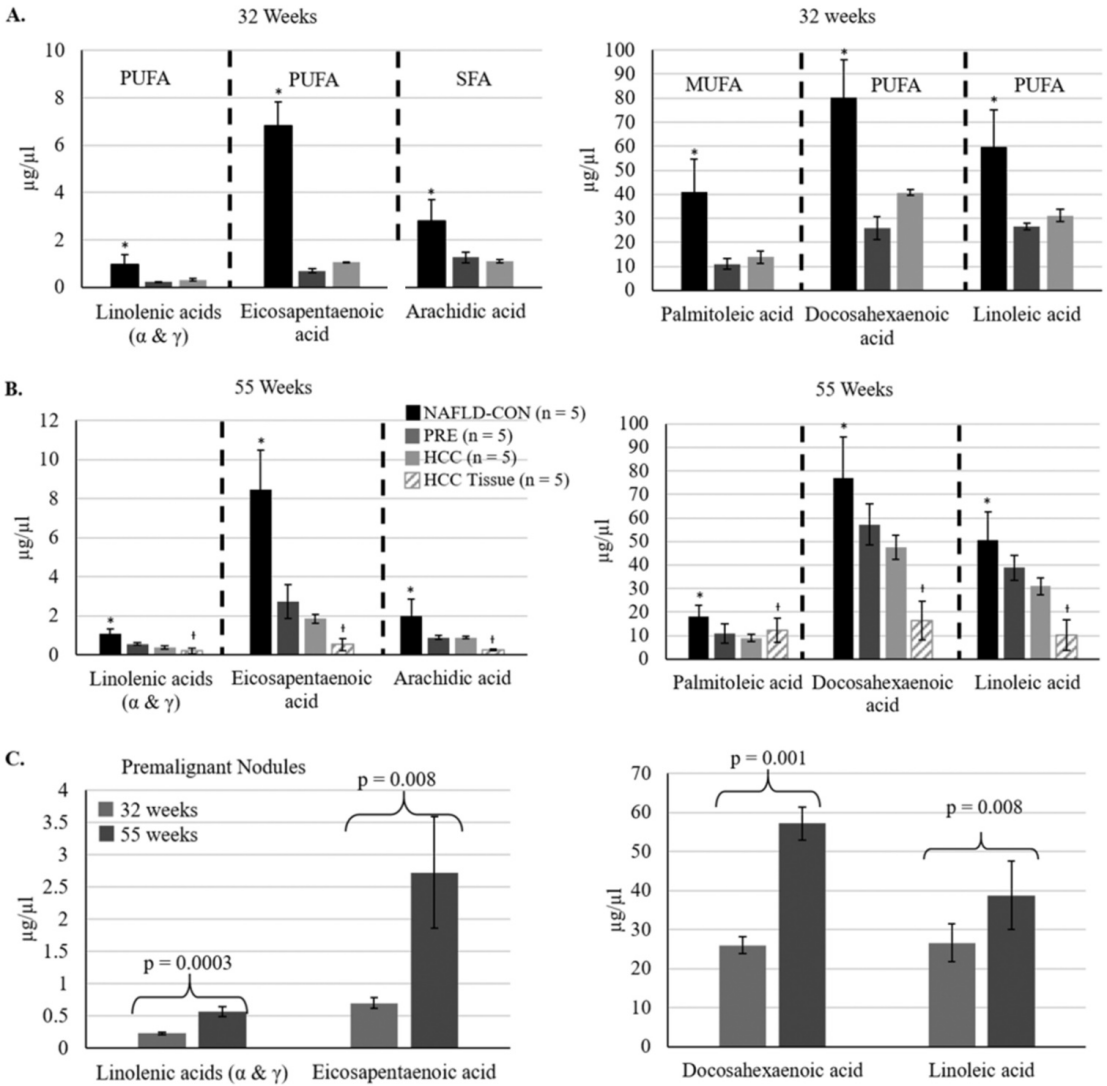
Fatty acid levels in mice fed the CD-HFSC diet with HCC and pre-malignant (PRE) nodules, and mice with NAFLD fed the control diet (NAFLD-CON). **A and B.** Mice with nodules (PRE and HCC) had significantly lower plasma levels of specific fatty acids than control mice with NAFLD at 32 and 55 weeks (* = p < 0.05). **C.** The levels of polyunsaturated fatty acids increased from 32 weeks to 55 weeks in mice with premalignant nodules. Polyunsaturated fatty acids = PUFA; monounsaturated fatty acids = MUFA; saturated fatty acids = SFA; +µg/mg.

We evaluated plasma levels of thirty fatty acids at 32 weeks of age and the study endpoint (55-56 weeks of age) (**Supplemental Table S2**). At 32 weeks 11 of the 30 fatty acids were reduced significantly in the plasma of mice fed the CD-HFSC diet (with pre-malignant and malignant lesions) compared to mice fed the control diet (with NAFL). 73% of those fatty acids were polyunsaturated fatty acids (PUFAs). At 55-56 weeks of age, 9 fatty acids were reduced significantly in the plasma of mice fed the CD-HFSC diet compared to mice fed the control diet. 67% of those fatty acids were PUFAs (**Supplemental Table S4**). There were no significant differences in the fatty acid levels between mice with HCC and mice with pre-malignant nodules; however, there was a further reduction in the levels of these fatty acids in mice with HCC compared to mice with pre-malignant lesions (**Figure 5**). Five fatty acids were significantly reduced in mice fed the CD-HFSC diet compared to mice fed the control diet at both time points: linolenic acid (α and γ), eicosapentaenoic acid (EPA), palmitoleic acid, docosahexaenoic acid (DHA), linoleic acid, and arachidic acid (**Figure 5A and B**). These same fatty acids significantly increased over time in mice with pre-malignant lesions (**Figure 5C**).

### Assessment of expression of genes related to control of PUFA levels

We conducted mRNA sequencing analysis to compare gene expression in livers of mice fed the control diet with NAFLD and mice fed the CD-HFSC diet with HCCs (**Table 2**). We found reduced levels of genes controlling desaturase expression such as sterol regulatory element-binding protein 1 (Srebf1), Srebp cleavage activating protein (Scap), fatty acid synthase (Fasn), and ATP citrate lyase (Acly) in HCCs compared to livers with NAFLD. In addition, we found reduced levels of genes affecting homocysteine levels such as cystatheione beta synthase (Cbs). We also found increased levels of Methylenetetrahydrofolate Dehydrogenase (NADP+ Dependent) 1 Like (Mthfd1l), which would result in higher levels of tetrahydrofolate (THF). With annotation of the differentially expressed genes (DEGs) to pathways, we found the highest percentage of DEGs to belong to metabolic pathways (14.6%), followed by 5.02% of DEGs belonging to cancer pathways, and 3.93% belonging to the PI3K-Akt signaling pathway when comparing NAFLD with HCC.

We also assessed protein expression of PPARα. PPARα contributes to the activation of desaturases that are the rate-limiting enzymes for PUFA conversion and hence are the main determinants of PUFA levels. In addition, it is implicated in lipid metabolism and inflammation responses. The expression of PPARα was lower in the tumors of mice fed the CD-HFSC diet and some of the pre-malignant nodules compared to NASH tissue in CD-HFSC fed mice and NAFLD tissue from mice fed the control diet (**Supplemental Figure 1**).

## Discussion

A diet deficient in choline and high in trans-fat/cholesterol/sucrose resulted in the development of NASH-HCC with high penetrance in lean mice. These mice, like many lean NASH patients do not exhibit glucose intolerance nor high levels of plasma cholesterol and triglycerides 21. Choline deficiency results in triglyceride retention in the liver since it impacts on the packaging and export of triglycerides through VLDL from the liver 22. These mice exhibited higher plasma levels of liver enzymes, indicating ongoing liver damage that is consistent with high fat diet-induced mitochondrial lipid metabolism, increased production of reactive oxygen species and consequent lipotoxicity 23.

Mice with lean NASH had the key feature of hepatocyte ballooning observed in human NASH. The livers of all mice with NASH exhibited hepatocyte ballooning, with 53.6% having a ballooning score of 2. The lean NASH-HCC model developed in this work presents several advantages to existing NASH-HCC models. It is the first lean NASH-HCC model that shows a high (60.7%) penetrance of HCC after the shortest duration (52 weeks) on the experimental diet. Another model that used a choline deficient/high fat diet showed development of HCC in non-obese mice with 27% penetrance after 60 weeks on the diet 17. Other non-obese HCC models involve use of a choline deficient diet or a choline deficient/ethionine supplemented diet, albeit in these models mice develop HCC after 80 weeks on the diet with 22% and 86% penetrance, respectively 18. The other non-obese model of NASH-HCC involves pTEN gene deletion and these mice develop HCC after 74-78 weeks on a chow diet with 66% penetrance 13. Other NASH-HCC models occur in the context of obesity and mice develop small HCCs with 60% penetrance after 52 weeks on a high trans-fat/sucrose/fructose diet or HCCs with 25% penetrance after 48 weeks on a choline deficient/high sucrose/fat diet 15,16. Of note, the aforementioned obese NASH-HCC models used the C57BL/6J strain, which has a polymorphism in the Nnt gene implicated in insulin response, as opposed the C57BL/6N strain we used in this study 24. We suggest that the C57BL/6N strain is ideal to use for a lean NASH-HCC model.

Deregulated lipogenesis is a feature of carcinogenesis. In our mice, mostly PUFAs showed lower plasma levels in mice with pre-malignant and malignant nodules compared to mice with fatty liver fed the control diet. Furthermore, some of these PUFAs increased over time in mice with pre-malignant nodules, suggesting they may be important for cancer inhibition. These PUFAs included linolenic acids (α and γ, ω-3 and ω-6, respectively), EPA (ω-3), DHA (ω-3), and linoleic acid (ω-6). These PUFAs have been previously shown to be involved in liver carcinogenesis. For example, γ linolenic acid has been shown to reduce cell proliferation and promote the generation of ROS and apoptosis in HCC cell lines 25. Furthermore, it had a chemo-protective effect against diethylnitrosamine-induced HCCs 26. EPA- and DHA-enriched diet fed mice were shown to be metabolically healthier with lower lipid and fatty acid biosynthesis as well as upregulation of PPARα and lipid catabolism 27. In our mice, lower plasma levels of these fatty acids occurred with tumor progression and lower levels of PPARα in the tumors (**Supplemental Figure 1**). Linoleic acid was the only fatty acid that was significantly reduced in human HCC tissue in a tissue metabolomics investigation of human HCC 28. Furthermore, a mouse model of NASH-HCC using mice with hepatocyte deletion of pTen also revealed reduction in linoleic and γ-linolenic acids in plasma and NASH tissue and further reduction of linoleic acid in HCC tissue 13.

One possible mechanism for lower PUFA production in our mice may be lower expression of PPARα in pre-malignant and HCC tissues, which may contribute to higher levels of inflammation and reduced production of PUFAs by desaturases (**Supplemental Figure 1**). More specifically, PPARα has been shown to be involved in the control of the desaturases (Δ5 and Δ6) in the liver that affect the production of these PUFAs 29. Furthermore, mRNA sequencing analysis revealed lower expression of genes implicated in desaturase expression such as Srebf1, Scap, Fasn, and Acly in HCCs compared to livers with NAFLD. This suggests that the production of PUFAs is suppressed by changes in gene expression during tumor progression. Interestingly, genes implicated in homocysteine metabolism such as Cbs were also downregulated in HCCs compared to NAFLD, which could result in higher homocysteine levels. PUFAs have been shown to modulate homocysteine levels, whereby PUFA supplementation results in reduction of homocysteine levels 30-32. This data suggests that reduction of PUFAs along with increase in homocysteine levels may be contributing to lean NASH-HCC progression. Elevated homocysteine levels have been previously suggested to promote hepatocarcinogenesis 33. EPA and DHA, which were reduced in our HCC-bearing mice, are considered to be the biosynthetic precursors of potent anti-inflammatory mediators such as resolvins and protectins, suggesting that their reduction may be permissive of a tumor-promoting inflammatory environment. Resolvin E (RvE) and resolvin D (RvD) series of resolvins are derived from EPA and DHA. Interestingly, resolvin D1 and resolving E1 were found to prevent liver injury and the progression from hepatitis to liver cancer through inhibition of NFκB in mice 34. Furthermore, resolvin D1 was shown to prevent epithelial mesenchymal transition and cancer stemness in HCC 35. Further supporting the link between PUFAs and inflammation, omega 3 PUFAs such as EPA and DHA have been shown to inhibit hepatocellular carcinoma cell growth by blocking inflammation mediators such as cyclooxygenase 2 (COX-2) 36.

Another observation from our mRNA sequencing analyses was that DEGs that belong to metabolic pathways were the most frequent (14.6%), followed by 5.02% of DEGs belonging to cancer pathways, and 3.93% belonging to the PI3K-Akt signaling pathway when comparing NAFLD with HCC. Interestingly, the PI3K-Akt signaling pathway has been linked to liver inflammation in HCC 37. Therefore, it is plausible that reduction of PUFAs may be contributing to increased inflammation in a PI3K-Akt pathway and NFκB dependent manner. On a final note, we also found increased levels of Mthfd1l, which would result in higher levels of THF. We suggest that increased production of THF would not be used for homocysteine remethylation but rather be used for nucleotide synthesis to support tumor cell proliferation. This would also contribute to the accumulation of homocysteine.

In conclusion, we present a novel lean NASH-HCC model using the C57BL/6N strain that shows high penetrance of HCC after 52 weeks on a choline deficient and high trans-fat/sucrose/cholesterol diet. Furthermore, our study shows for the first time that certain PUFAs (linolenic acids (α and γ, ω-3 and ω-6, respectively), eicosapentanoic acid (ω-3), docosahexanoic acid (ω-3), and linoleic acid (ω-6)) show reduced levels in lean NASH-HCCs and increasing levels in mice with pre-malignant lesions suggesting their anti-carcinogenic properties. We suggest further study of the mechanisms driving lower PUFA levels as well as the molecular and cellular consequences of lower PUFA production. We also suggest to consider using PUFA supplementation as a way to inhibit HCC formation and PUFA levels as potentially biomarkers for lean NASH-HCC. This has been suggested for colorectal cancer (CRC). A meta-analysis and systematic review of CRC and blood PUFA levels has shown that high blood n-3 PUFA levels are inversely associated with colorectal cancer risk and high intake of these PUFAs has been found to be associated with lower colorectal cancer risk 38. Finally, consumption of n-3 fatty acids has been shown to be inversely associated with HCC in a population-based prospective cohort study of Japanese individuals 39.

## Supplementary Material

Supplementary figures and tables.

## Figures and Tables

**Table 1:** Frequency of fibrosis stages 1-3, NAFL, and NASH in mice fed the control diet and mice fed the CD-HFSC diet at 55-56 weeks of age. All mice fed the CD-HFSC diet developed NASH but none developed cirrhosis

	Fibrosis 1	Fibrosis 2	Fibrosis 3	NAFL	NASH	Regenerative Nodules	Dysplastic Nodules	HCC
CD-HFSC diet	50% (14/28)	42.9% (12/28)	7.1% (2/28)	0% (0/28)	100% (28/28)	92.9% (26/28)	89.3% (25/28)	60.7% (17/28)
Control diet	0% (0/15)	0% (0/15)	0% (0/15)	53.3% (8/15)	0% (0/15)	0% (0/15)	0% (0/15)	0% (0/15)

**Table 2:** DEGs related to desaturase expression and homocysteine levels when comparing livers with NAFLD from control mice to HCCs from mice fed the CD-HFSC diet

Gene	Log2 fold change	Adj P-value
*Cbs*	-1.807900844	6.25 × 10^-6^
*Srebf1*	-2.650504371	1.39 × 10^-10^
*Scap*	-2.36427007	9.78 × 10^-10^
*Mthfd1l*	2.830242531	2.25 × 10^-6^
*Fasn*	-3.549853812	6.80 × 10^-10^
*Acly*	-3.430667537	5.87 × 10^-8^
