# Supplementary material for: Reduction of Polyunsaturated Fatty Acids with Tumor Progression in a Lean Non-Alcoholic Steatohepatitis-Associated Hepatocellular Carcinoma Mouse Model
Source: J Cancer. 2020 Jul 20;11(19):5536–46. doi: 10.7150/jca.48495 (PMC7477439; doi:10.7150/jca.48495)
Supplement: Supplementary file 1 — Supplementary figures and tables. [file jcav11p5536s1.pdf]

Supplemental Table S1. The table shows the composition of the control diet and the choline deficient high trans-fat/sucrose/cholesterol diet (CD-HFSC). *Fat is mostly trans-fat from Primex.

Nutrient	Control Diet	CD-HFSC Diet
Fat (% kcal)	10	44.9*
Protein (% kcal)	20	20
Carbohydrate (% kcal)	70	35.1
Sucrose (% kcal)	0	29.1
Cholesterol (% g)	0	2
Choline (% g)	0.2	0
**Total Calories**	**4,057**	**4,057**

Supplemental Table S2. The table shows the thirty fatty acids investigated in this study.

Fatty Acids		
Saturated fatty acids (SFA)	Monounsaturated fatty acids (MUFAs)	Polyunsaturated fatty acids (PUFAs)
Lauric acid (C12)<br>Myristic acid (C14)<br>Pentadecanoic acid (C15)<br>Palmitic acid (C16)<br>Margaric acid (C17)<br>Stearic acid (C18)<br>Arachidic acid (C20)<br>Behenic acid (C22)<br>Lignoceric acid (C24)<br>Cerotic acid (C26)	Palmitoleic acid (16:1)<br>Oleic acid (18:1)<br>Eicosenoic acid (20:1)<br>Mead acid (20:3)<br>Heptadecenoic acid<br>Erucic acid (22:1)<br>Nervonic acid (24:1)	Linoleic acid (ω-6) (18:2)<br>Linolenic acids α (ω-3) & γ (ω-6) (18:3)<br>Stearidonic acid (ω-3) (18:4)<br>Eicosadienoic acid (ω-6) (20:2)<br>Dihomo-γ-linolenic acid (ω-6) (20:3)<br>Arachidonic acid (ω-6) (20:4)<br>Eicosapentaenoic acid (ω-3) (20:5)<br>Docosadienoic acid (ω-6) (22:2)<br>Docosatrienoic acid (ω-3) (22:3)<br>Adrenic acid (ω-6) (22:4)<br>Clupanodonic acid (ω-3) (22:5)<br>Osbond acid (ω-6) (22:5)<br>Docosahexaenoic acid (ω-3) (22:6)

Supplemental Table S3. Gross observations of mice fed the control diet and the CS-HFSC diet upon necropsy. Mice fed the CD-HFSC diet had enlarged livers, significantly higher liver to body weight ratio and enlarged spleens compared to mice fed the control diet.

	Control Diet	CD-HFSC Diet	p value
Average liver weight	1.57 g (+/- 0.16 g)	4.41 g (+/- 0.79 g)	1.56×10^{-16}
Average liver size	598.33mm^2 (+/-115.17 mm^2)	1002.56 mm^2 (+/- 1181.29 mm^2)	2.63×10^{-9}
Average spleen weight	0.11 g (+/- 0.06 g)	0.24 g (+/- 0.06 g)	2.99×10^{-18}
Liver weight : body weight	0.04	0.14	2.44×10^{-7}
Max nodule number	0	24	
Min nodule number	0	1	
Average nodule #	0	11	
Median nodule #	0	11	
Max nodule size	0	600 mm^2	
Min nodule size	0	1 mm^2	
Average nodule size	0	44.20 mm^2	
Median nodule size	0	4.5 mm^2	
Regenerative nodules	0	Max: 8<br>Average: 3.6	
Dysplastic nodules	0	Max: 8<br>Average: 3.6	
Average HCC	0	Max: 4<br>Average: 1.1	

Supplemental Table S4. The tables shows a list of fatty acids whose levels were reduced at 32 or 55 weeks of age. No differences were observed between mice with pre-malignant nodules and mice with HCC. Six fatty acids (italic) were significantly reduced at both 32 and 55 weeks. Polyunsaturated fatty acids = PUFA; monounsaturated fatty acids = MUFA; saturated fatty acids = SFA

**Plasma Fatty Acid Reduction at 32 weeks**	**p value**
Eicosapentaenoic acid (PUFA)	3.82×10^{-5}
Docosahexaenoic acid (PUFA)	0.002268
Arachidonic acid (PUFA)	0.007774
Docosapentaenoic acid (n-3; PUFA)	0.009972
Palmitoleic acid (MUFA)	0.016617
Linoleic acid (PUFA)	0.019494
Docosadienoic acid (PUFA)	0.020973
Linolenic acids (α and γ) (PUFA)	0.030345
Arachidic acid (SFA)	0.035144
Dihomo-g-linolenic acid (PUFA)	0.041485
Heptadecenoic acid (MUFA)	0.045548
**Plasma Fatty Acid Reduction at 55 weeks**	**p value**
Eicosapentaenoic acid (PUFA)	1.58 × 10-5
Linolenic acids (α and γ) (PUFA)	0.000178
Oleic acid (MUFA)	0.000399
Docosapentaenoic acid (n-6; PUFA)	0.001066
Stearidonic acid (PUFA)	0.00299
Docosahexaenoic acid (PUFA)	0.011437
Arachidic acid (SFA)	0.012421
Palmitoleic acid (MUFA)	0.012511
Linoleic acid (PUFA)	0.014917

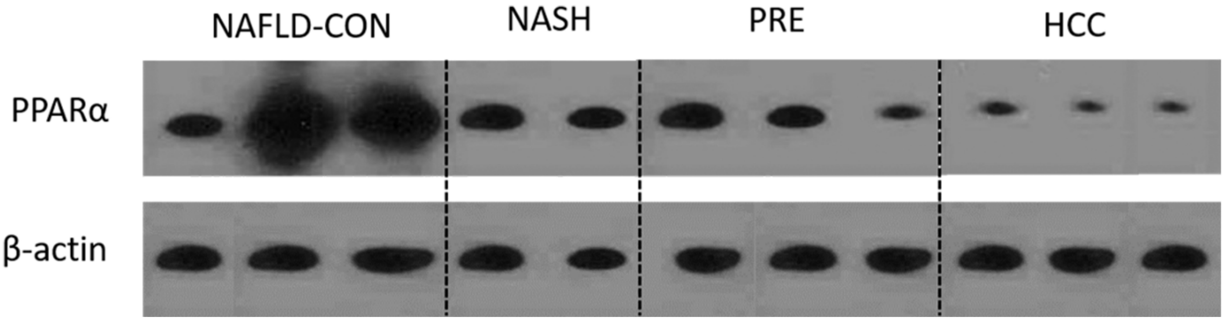

Supplemental Figure 1. PPARα expression in NASH tissue, HCC and pre-malignant (PRE) nodules from mice fed the CD-HFSC diet, and NAFLD tissue from mice fed the control diet (NAFLD-CON). Western blot analyses were conducted using PPARα-specific antibodies and β-actin antibodies (loading control). Overall PPARα protein expression is lower in NASH tissue, pre-malignant lesions and tumors of mice fed the CD-HFSC diet compared to the NAFLD tissue from mice fed the control diet. The levels of PPARα protein are lower in HCC compared to pre-malignant lesions and NASH tissue.
